# Anions for Near-Infrared Selective Organic Salt Photovoltaics

**DOI:** 10.1038/s41598-017-16539-3

**Published:** 2017-11-27

**Authors:** Christopher J. Traverse, Margaret Young, John Suddard-Bangsund, Tyler Patrick, Matthew Bates, Pei Chen, Brian Wingate, Sophia Y. Lunt, Annick Anctil, Richard R. Lunt

**Affiliations:** 1 Department of Chemical Engineering and Materials Science, Michigan State University, East Lansing, MI 48824 USA; 2 Department of Mechanical Engineering, Michigan State University, East Lansing, MI 48824 USA; 3 Department of Biochemistry and Molecular Biology, Michigan State University, East Lansing, MI 48824 USA; 4 Department of Civil and Environmental Engineering, Michigan State University, East Lansing, MI 48824 USA; 5 Department of Physics and Astronomy, Michigan State University, East Lansing, MI 48824 USA

## Abstract

Organic molecular salts are an emerging and highly tunable class of materials for organic and transparent photovoltaics. In this work, we demonstrate novel phenyl borate and carborane-based anions paired with a near-infrared (NIR)-selective heptamethine cation. We further explore the effects of anion structures and functional groups on both device performance and physical properties. Changing the functional groups on the anion significantly alters the open circuit voltage and yields a clear dependence on electron withdrawing groups. Anion exchange is also shown to selectively alter the solubility and film surface energy of the resulting molecular salt, enabling the potential fabrication of solution-deposited cascade or multi-junction devices from orthogonal solvents. This study further expands the catalog and properties of organic salts for inexpensive, and stable NIR-selective molecular salt photovoltaics.

## Introduction

Anion exchange with near-infrared (NIR)-selective cyanine (Cy^+^) heptamethine cations enables facile tuning of the frontier orbital energies, the interface gap (the difference in the donor highest occupied molecular orbital and the acceptor lowest unoccupied molecular orbital) between the salt donor and fullerene (C_60_) acceptor, and thus the open circuit voltage (*V*_*oc*_)^1–3^. This allows for the fabrication of Cy-based devices that approach the excitonic voltage limit. Because the Cy^+^ cation is primarily responsible for optical absorption, exchanging the anion does not significantly affect the spectral range or magnitude of the extinction coefficient (see Figure S1). Absorption is instead tuned via conjugation^4^ of the cation molecule which can enable efficient NIR photoresponse at wavelengths of up to 1600nm^3^, ideal for applications in NIR-selective transparent photovoltaics (TPVs)^5,6^. While efficiencies have reached nearly 4% for this class of materials^7^, and properties such as the highest occupied molecular orbital (HOMO) and exciton diffusion have been linked to anion selection^2^, very little is known about the full range of tunability provided by anion pairing alone.

In this work, we demonstrate alternative anions from two families, phenyl borates and carboranes, to determine the effects of the electron withdrawing groups and anion size on the performance and energy level alignment of devices utilizing the same Cy^+^ cation. We select anions from the family of phenyl borates because they afford large-sized anions which have previously been correlated with high exciton diffusion lengths (*EDL*s)^2^. Salt-based devices with tetrakis(pentafluorphenyl)borate (TPFB) anions greatly out-performed equivalent devices with smaller anions due to a high *EDL* and larger interface gap.

Carboranes are highly stable carbon-boron molecular clusters that have emerged recently for use as superacids^8,9^ and have been shown to serve as extremely low coordinating anions for use in organic salts, with demonstrated applications in fabricating molecular wires^10^ and electrochemical capacitors^11^. They have also been employed in manipulating the band gap of emission layers to tune the emission color of phosphorescent organic light emitting diodes^3,12^. Because of their low coordination, these anions may yield a path toward exceptionally stable organic salt PVs.

## Results and Discussion

**Figure 1:**
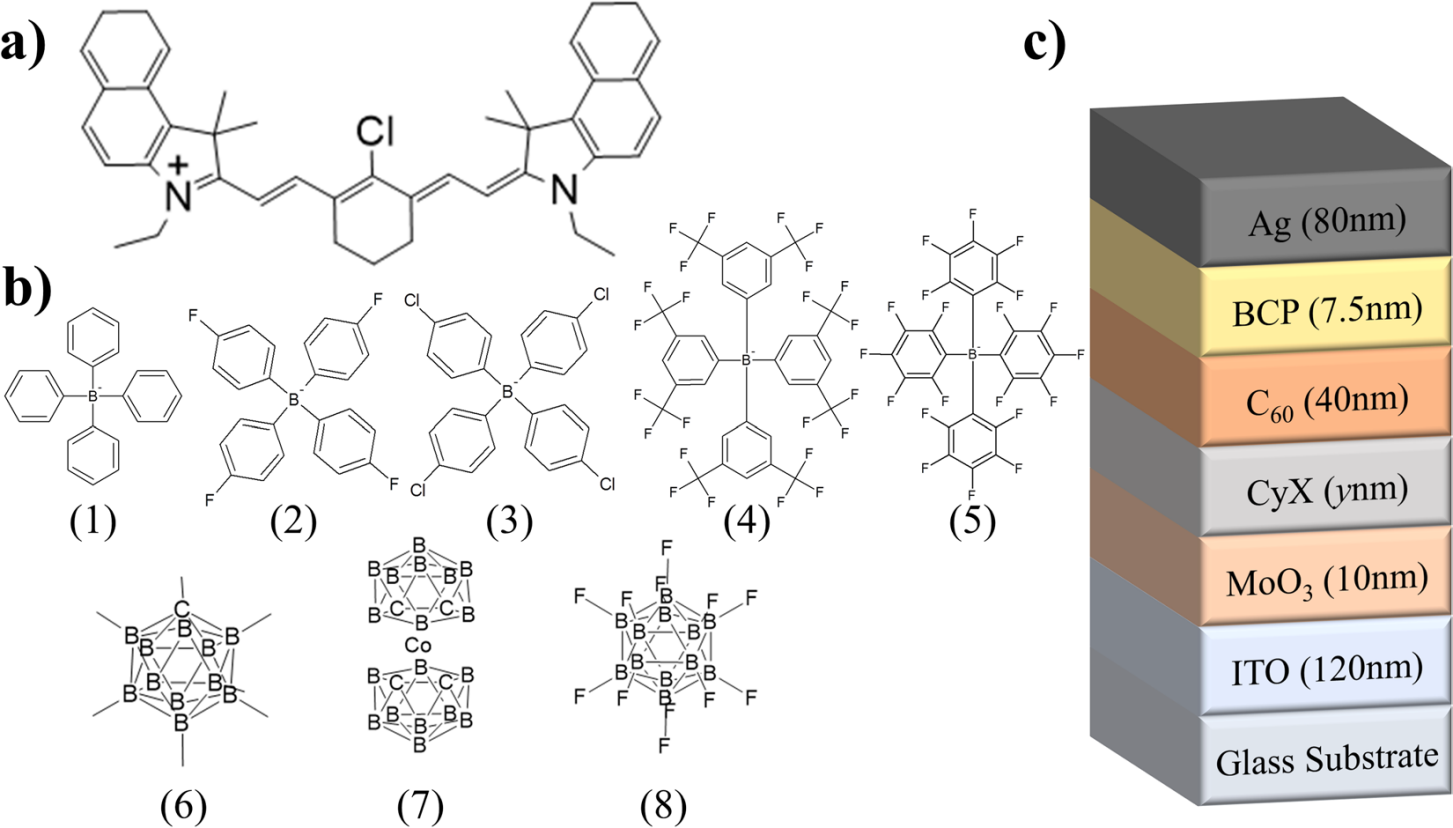
Molecular structures and experimental device architecture (**a**) Chemical structure of the Cy^+^ heptamethine cation. (**b**) Chemical structures for tetraphenylborate (1) tetrakis(4-fluorophenyl)borate (FPhB^−^) (2), tetrakis(4-chlorophenyl)borate (ClPhB^−^) (3), tetrakis[3,5-bis(trifluoromethyl)phenyl]borate (TFM^−^) (4), tetrakis(pentafluorophenyl)borate (TPFB^−^) (5), CB_11_H_12_ (CBH^−^) (6), C_4_B_18_Co (CoCB^−^) (7), and B_12_F_12_ (FCB^2−^) (8) anions. (**c**) Illustration of the photovoltaic device stack utilized in this study.

The phenyl borates investigated include tetraphenylborate (CyPhB), tetrakis(4-fluorophenyl)borate (CyFPhB), tetrakis(4-chlorophenyl)borate (CyClPhB), tetrakis[3,5-bis(trifluoromethyl)phenyl]borate (CyTFM), and CyTPFB (control device) while the carboranes include CB_11_H_12_ (CyCBH), C_4_B_18_Co (CyCoCB), and B_12_F_12_ (Cy_2_FCB). The heptamethine cation and all anions are shown in Fig. 1a,b, respectively. Cy salts were prepared by anion exchange of the parent CyI compound (see Methods for full procedures). Mass spectrometry was utilized to confirm the yield for each cation-anion pairing (Figures S2 and S3). Photovoltaic devices were fabricated in the following architecture (Fig. 1c): indium tin oxide (ITO) (120 nm) / MoO_3_ (10 nm) / CyX (*y* nm) / C_60_ (40 nm) / bathocuproine (BCP) (7.5 nm) / Ag (80 nm), where X is the anion paired with Cy^+^ and *y* is the donor layer thickness. Cy donor layers were deposited from dimethylformamide (DMF) or chlorobenzene (CB) under nitrogen while all other layers were thermally deposited under vacuum. Cy layer thicknesses were controlled by varying the solution concentrations between 2–12 mg/mL to determine the effects of energy band bending on the interface gap.

**Figure 2:**
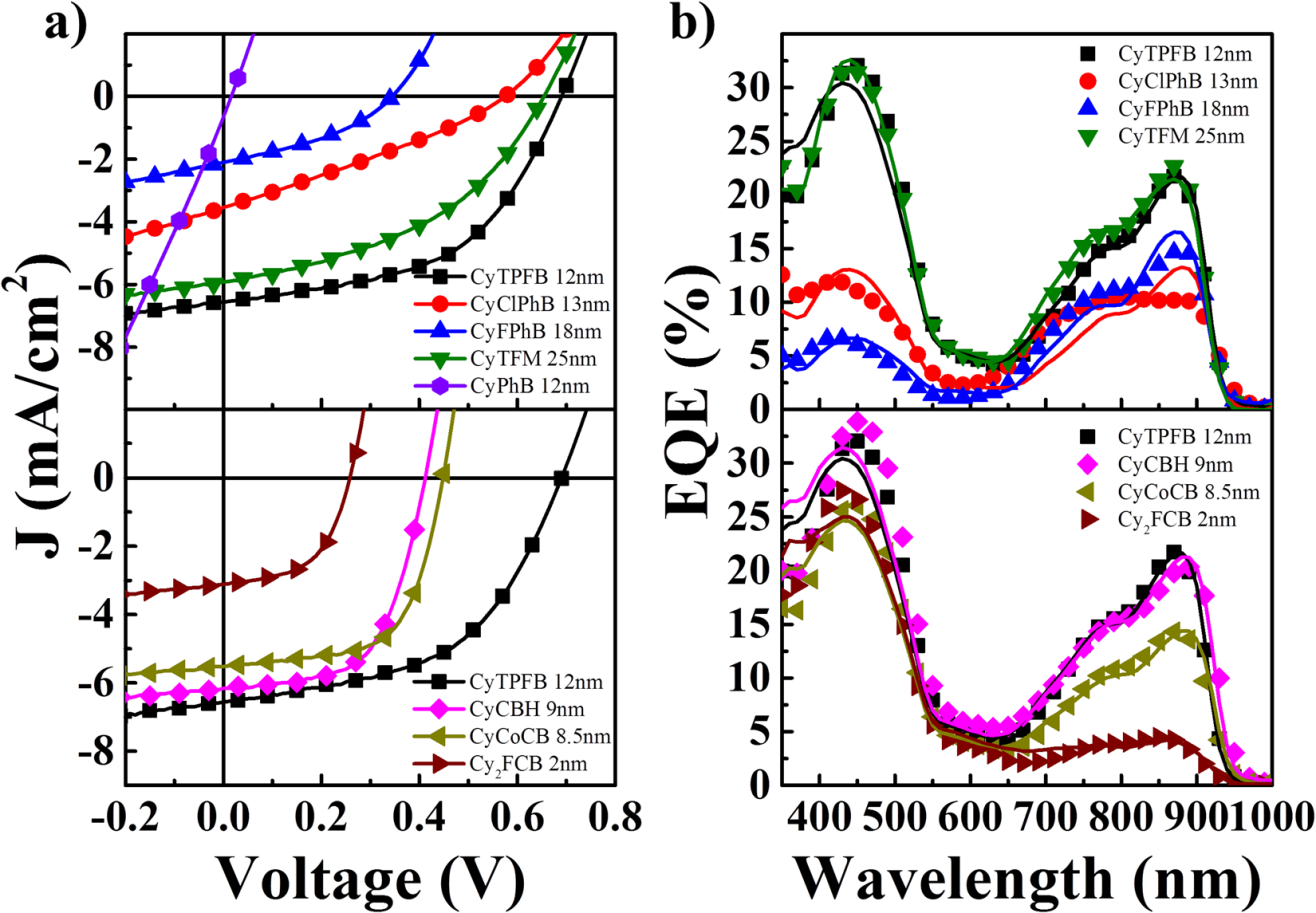
Photovoltaic device data. (**a**) Current density-voltage (*J-V*) and (**b**) external quantum efficiency (*EQE*) characteristics for best devices. Symbols and lines in (**b**) correspond to measured and fitted *EQE* data respectively, the latter of which was generated when fitting spectra for exciton diffusion lengths shown in Table 1.

Current (*J*)-voltage (*V*) and external quantum efficiency (*EQE*) characteristics of the devices utilizing each anion are shown in Fig. 2, with average performance metrics for the optimum thicknesses and calculated donor *EDL*s shown in Table 1. *EDL*s were extracted from *EQE* data with a combined diffusion and optical interference model using least squares regression^13^. CyTPFB exhibits the overall highest power conversion efficiency (*PCE*) due to high combined short circuit current density (*J*_*sc*_), *V*_*oc*_, and fill factor (*FF*). CyTFM exhibits similar *PCE* to CyTPFB because of high *J*_*sc*_ and *V*_*oc*_ likely due to a similar molecular size and HOMO respectively. On average, CyCoCB exhibits the best *PCE* of the carboranes due to high *FF* and *V*_*oc*_, though CyCBH exhibits the best *J*_*sc*_ (and *EQE*) similar to that of CyTPFB. Each carborane exhibits a high *FF* indicating low series and high shunt resistance, however the *V*_*oc*_s are considerably lower than CyTPFB and the rest of the functionalized phenyl borates. High *J*_*sc*_s can be attributed to improved *EDL*s over the smaller phenyl borates, though the mechanism for this improvement is unclear.

**Table 1:** Device performance parameters.

Donor (Thickness)	J_sc_ (mA/cm^2^)	V_oc_(V)	FF	PCE(%)	NIR EQE (%)	EDL(nm)
CyTPFB (12 nm)	5.6 ± 0.6	0.66 ± 0.03	0.57 ± 0.04	2.1 ± 0.1	21.7	4.3 ± 0.3
CyClPhB (13 nm)	3.5 ± 0.3	0.59 ± 0.05	0.29 ± 0.01	0.59 ± 0.06	10.5	2.6 ± 0.2
CyFPhB (18 nm)	2.1 ± 0.2	0.36 ± 0.01	0.36 ± 0.01	0.27 ± 0.03	14.9	3.4 ± 0.1
CyTFM (25 nm)	5.9 ± 0.6	0.69 ± 0.04	0.42 ± 0.01	1.7 ± 0.2	20.7	7.5 ± 0.4
CyPhB (12 nm)	0.65 ± 0.06	0.02 ± 0.01	0.25 ± 0.01	0	0	—
CyCBH (9 nm)	5.7 ± 0.3	0.42 ± 0.01	0.56 ± 0.03	1.3 ± 0.1	20.4	3.6 ± 0.3
CyCoCB (8.5 nm)	5.2 ± 0.3	0.45 ± 0.01	0.61 ± 0.01	1.4 ± 0.1	14.3	2.5 ± 0.2
Cy_2_FCB (2 nm)	3.0 ± 0.2	0.27 ± 0.01	0.53 ± 0.01	0.42 ± 0.04	4.4	1.0 ± 0.1

Average performance parameters for optimum donor thicknesses, donor (near-infrared) external quantum efficiency (*EQE*) peak values, and exciton diffusion lengths (*EDL*s) calculated from *EQE* data in Fig. 2.

Ultraviolet photoelectron spectroscopy (UPS) data and estimated energy levels for each salt are shown in Figures S4 and S5. A high degree of fluorination on the anion is correlated with a deepening of the HOMO and lowest unoccupied molecular orbital (LUMO) of the salt donor^2,3^. Electron withdrawing groups consisting of electronegative atoms such as fluorine on the anion may induce a molecular dipole interaction (analogous to an interface dipole) between the anion and cation, shifting the HOMO and LUMO of the collective salt away from the vacuum level. The lack of fluorination on the PhB anion yields a low ionization energy that results in a nearly negligible interface gap with C_60_. This explains why the CyPhB devices exhibit little *J*_*sc*_ and *V*_*oc*_ < 0.1 V and high leakage current (Figure S6) across all donor thicknesses. The higher *V*_*oc*_s exhibited by the other salts can then be attributed to the electronegativities of atoms in the electron withdrawing groups, with the highest *V*_*oc*_s exhibited by CyTFM and CyTPFB owing to high degrees of distributed fluorination. This range of anions thus allows roughly 1 eV modulation of the HOMO.

**Figure 3:**
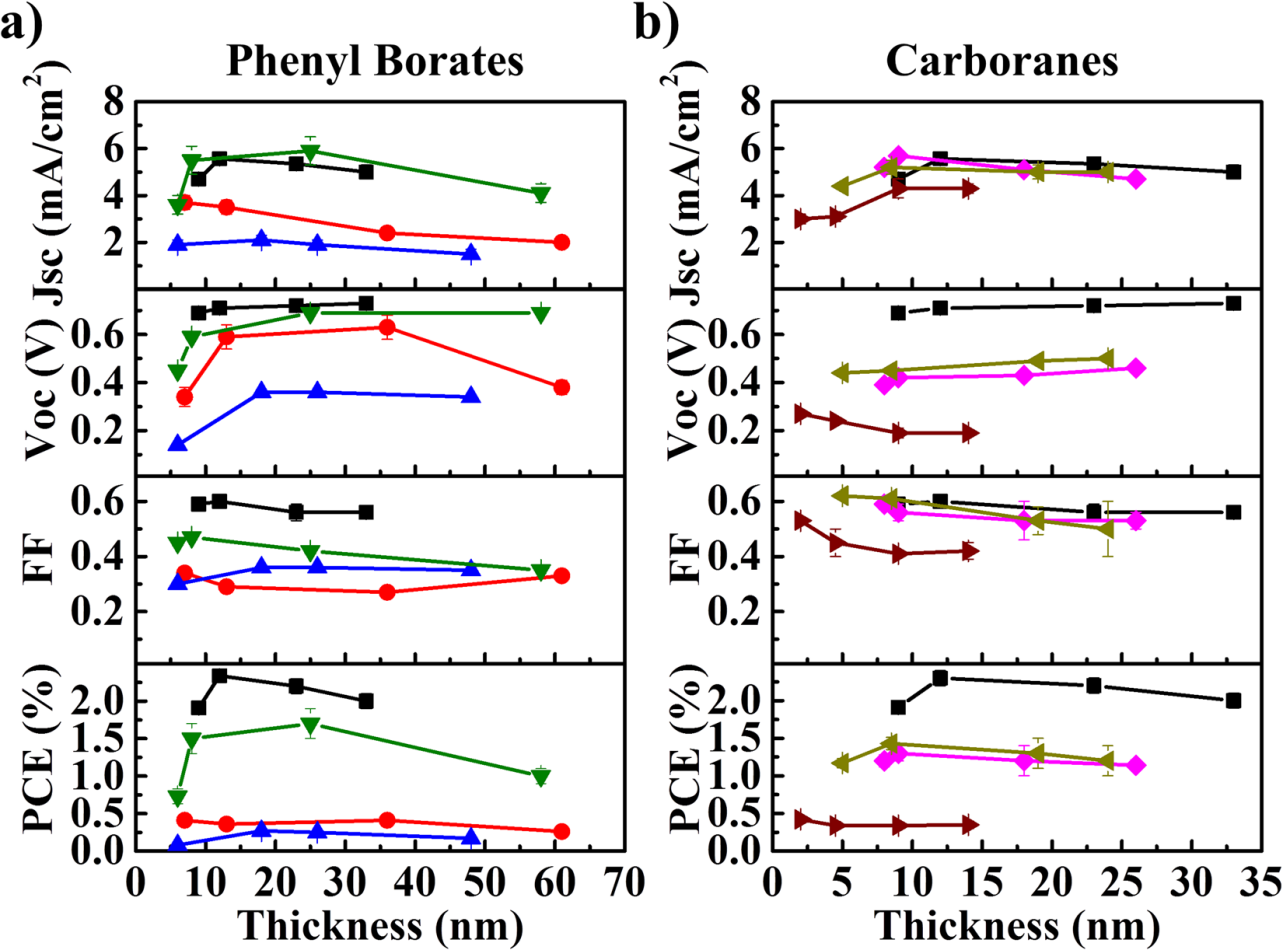
Performance thickness dependence. Thickness dependent performance data for salt devices with (**a**) phenyl borate and (**b**) carborane anions. The symbols and colors correspond to the anions in Fig. 2.

Thickness dependent performance data for the phenyl borates and carboranes are shown in Fig. 3. All anions follow a similar trend in *J*_*sc*_ with respect to thickness, where enhanced optical absorption efficiency yields a modest *J*_*sc*_ improvement as thickness is increased from 5 nm. As thickness is further increased beyond 20 nm, the optical absorption is offset by losses in exciton diffusion and carrier collection efficiencies as thickness exceeds the exciton diffusion and carrier collection lengths. Carrier collection loss is also mirrored by losses in the *FF*. The *J*_*sc*_ trend is correlated with the thickness dependence observed in the salt donor (NIR) portion of the *EQE* data (Figure S7). The *EQE* roll-off is determined largely by the *EDL*, where a high *EDL* as calculated for CyTFM yields a more gradual roll-off with increasing thickness. Other anions exhibit higher rates of *EQE* loss with increasing thickness indicative of either their shorter *EDL*s or charge collection lengths.

**Figure 4:**
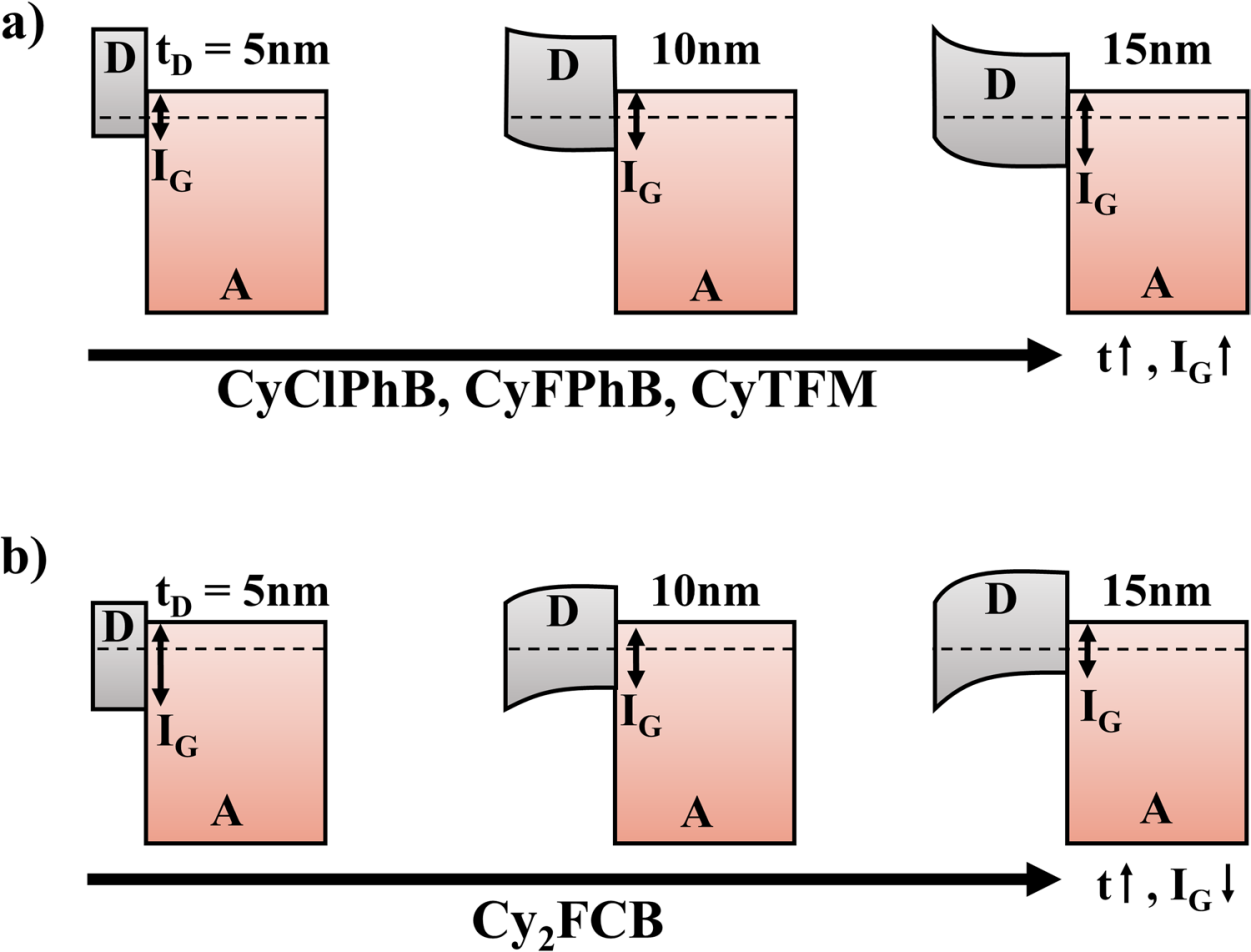
Anion band bending. Schematic donor-acceptor (D-A) band structures illustrating changes in interface gap (I_G_) as a function of donor thickness (t_D_). Anions given in (**a**) yield increasing I_G_ with increasing thicknesses while Cy_2_FCB yields the opposite trend illustrated in (**b**).

Most anions follow a similar voltage trend in which *V*_*oc*_ improves as thickness is increased from 5 nm and levels off before recombination losses begin to dominate at high thicknesses for CyClPhB and CyFPhB. CyCBH, CyCoCB, and CyTPFB show more limited *V*_*oc*_ trends as a function of thickness, and Cy_2_FCB exhibits an opposing voltage trend where *V*_*oc*_ is maximized at the lowest thicknesses. Atomic force microscopy (AFM) and cross-sectional scanning electron microscopy (SEM) data, shown in Figures S8 and S9, indicate smooth donor/acceptor interfaces. Although there may be some intermixing between the salts and C_60_ shown in the SEM images as evidenced by charging near the interface, this appears to occur over a distance of ~2 nm and is thus not likely to impact the *V*_*oc*_ of devices. It is important to note that anion diffusion from other molecular salts into C_60_ has been observed for small halide ions^14^ and while this process could potentially affect the *V*_*oc*_ through changes in the interface gap, it is reported to occur over several weeks and is thus unlikely to affect newly fabricated devices with much larger counterions. The more pronounced voltage trends exhibited by CyClPhB, CyFPhB, CyTFM, and Cy_2_FCB are instead caused by band bending of the salt donors and concomitant changes in the interface gap as illustrated in Fig. 4 and as seen in previous work with other anions^3^. Other anions show only modest changes in *V*_*oc*_ with thickness which are instead attributed to increased series resistance. Since the FCB anion carries a −2 charge, Cy_2_FCB layers contain higher packing densities of Cy^+^ cations yielding lower *J*_*sc*_ and *V*_*oc*_ likely from Coulombic scattering interactions with FCB.

While CyTPFB shows no appreciable degradation, CyFPhB and CyClPhB devices show a surprising lack of photostability compared to all other anions (see Figure S10). *J*_*sc*_ diminishes by approximately 30% for CyFPhB and 35% for CyClPhB, while *V*_*oc*_ diminishes by around 35% for both architectures after less than several minutes of direct illumination in air or under epoxy packaged in a nitrogen environment. This suggests that the rapid degradation is not due solely to air exposure, although parts per million of oxygen could be absorbed into the active films prior to packaging even in a nearly pure nitrogen environment. Photoinduced reactive oxygen species, including superoxide radical and singlet oxygen (formed from intersystem crossings to adsorbed oxygen molecules)^15^ are well-known degradation mechanisms in organic PVs due to their tendency to react with and photobleach active materials. Additionally, carbonyl groups formed from singlet oxygen interactions are known to be efficient exciton quenchers^16^ and further compound device performance losses. Singlet oxygen formation is likely if there is a favored intersystem crossing from the singlet to triplet state of the organic material. However, because the triplet resides on the cation and the overall intersystem crossing rate is not likely to be impacted by the presence of different anions, it is more likely electron or hole injection to form superoxide species that result in degradation. The formation of superoxide species has been proposed elsewhere^17^ as a charge transfer process in competition with exciton recombination and charge carrier extraction provided that the HOMO or LUMO of the donor or acceptor is closer to the vacuum level than the ground state of adsorbed oxygen. Large ionization energies can thus act as barriers against superoxide formation. We therefore attribute the rapid degradation of CyFPhB and CyClPhB to low ionization energies compared to the other salts due to lower concentrations of intramolecular polar bonds^18^ and electron withdrawing groups.

**Figure 5:**
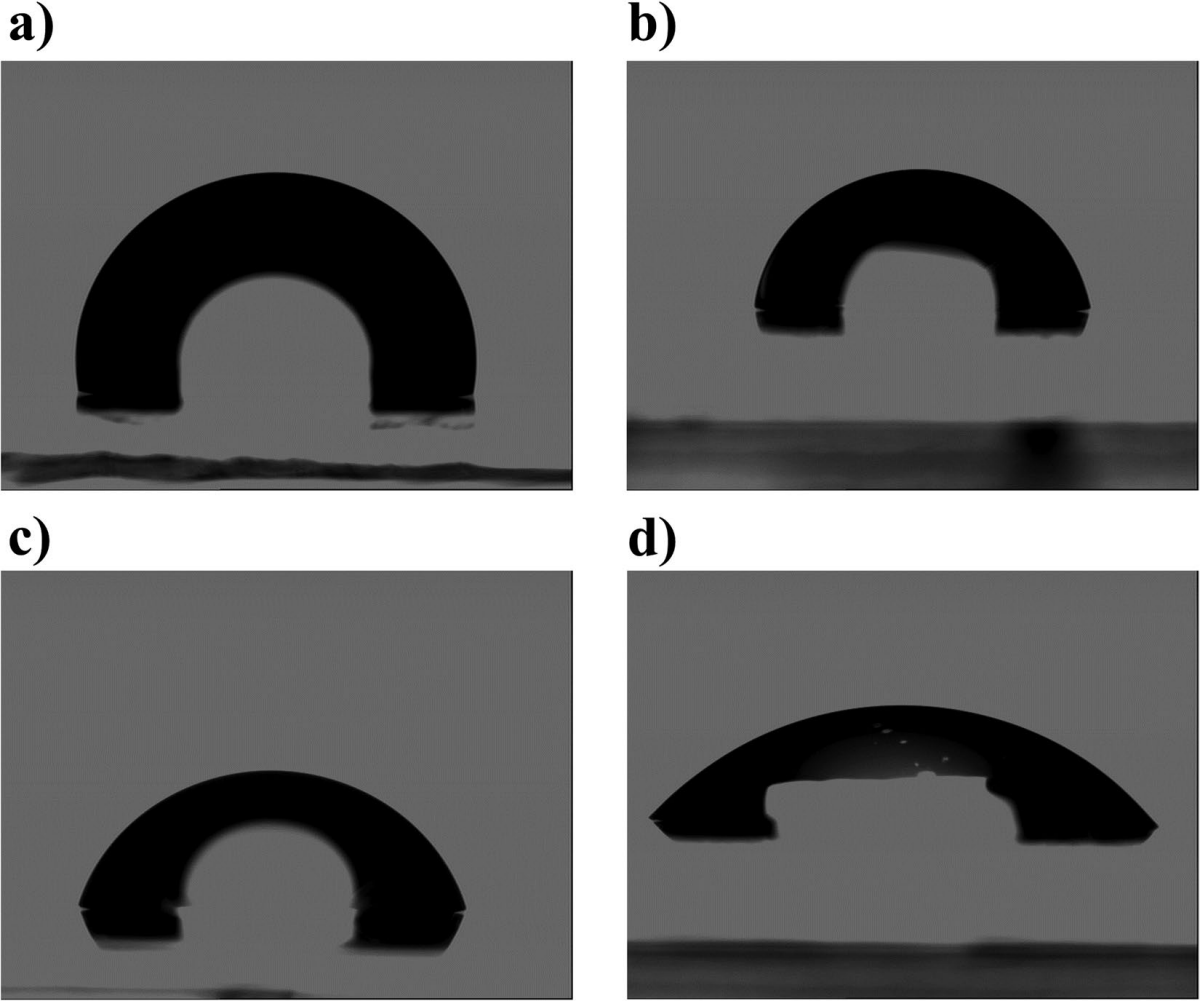
Tuning hydrophobicity. Photographs of water droplets on 50 nm films of (**a**) CyTPFB, (**b**) CyCoCB, (**c**) CyCBH, and (**d**) CyTFM (shown in order of decreasing contact angle) illustrate the ability to tune the hydrophobicity of the salt via anion exchange.

Contact angles, calculated surface energies, and solubilities for a range of anions are listed in Table 2. From the water contact angle data, we observe that the anion has a pronounced effect on the hydrophobicity of the salt film, where the water contact angle can be increased from 70 ± 2° (weakly hydrophilic) for CyCBH to 99.8 ± 0.4° (hydrophobic) for CyTPFB. Representative photographs of water droplets on various salts used to measure the water contact angles are shown in Fig. 5. It should be noted that all the salts investigated here are amorphous with little surface roughness. This ability to tune the surface energy with the donor layer may also be beneficial to overall device stability in the future and improve the flexibility in processing multi-layer devices.

**Table 2:** Surface energy and solubility data.

Salt	Water Angle (Degrees)	DIM Angle (Degrees)	Surface Energy (mN/m)	Solubility in CB (mg/mL)	Solubility in DMF (mg/mL)	Solubility in Water (mg/mL)
CyTPFB	99.8 ± 0.4	52 ± 4	38 ± 3	2.4	10	7 × 10^–5^
CyClPhB	76 ± 3	8.7 ± 0.6	52 ± 4	7.7	10	3 × 10^–5^
CyFPhB	72.9 ± 0.5	6 ± 1	51 ± 9	21	10	2 × 10^–5^
CyTFM	58 ± 7	50 ± 4	43 ± 5	19	10	1 × 10^–4^
CyPhB	77 ± 3	8 ± 2	50 ± 10	0.45	6.3	2 × 10^–5^
CyCBH	70 ± 2	7.2 ± 0.7	51 ± 5	0.28	10	2 × 10^–5^
CyCoCB	80 ± 1	13 ± 2	53 ± 8	0.27	10	2 × 10^–5^
Cy_2_FCB	76.3 ± 0.4	24.6 ± 0.4	47 ± 0.8	0.023	10	4 × 10^–5^
CyI	71 ± 2	0	55 ± 5	1	12	7 × 10^–5^
CyPF_6_	75 ± 4	25 ± 2	47 ± 3	2	9	5 × 10^–5^

Water and diiodomethane (DIM) contact angles, surface energies, and solubilities in chlorobenzene (CB), dimethylformamide (DMF), and water measured for salt films utilizing selected anions from ref.^2^ and the phenyl borate and carborane anions from this study.

The anion can also significantly impact the solubility in particular solvents. For example, the anion plays a significant role for dissolution in CB but very little role in water or DMF. Phenyl borate solubilities in CB are reduced from 21 mg/mL for CyFPhB to 0.45 mg/mL for CyPhB, while those of the carboranes are approximately equal ( ≤ 0.28 mg/mL). CyI and CyPF_6_ from previous work^2^ also exhibit low solubilities ≤ 2 mg/mL. The phenyl borates exhibit higher solubilities in CB than the other salts likely due to similarly structured phenyl groups of the anion enabling higher miscibility. Solubility differences in CB across the phenyl borates can be explained by differences in polarity in the phenyl groups; CyFPhB is especially soluble with the single fluorine on the phenyl group compared to the non-functionalized phenyl rings on CyPhB, which are more non-polar. Solubilities in DMF are consistently about 10 mg/mL across all anions including the carboranes, while those in water are quite low (<1 × 10^−4^ mg/mL) for both phenyl borates and carboranes. The molecular structures of these solvents are less similar to those of the anions. The solubility differences between DMF and water might be attributed to differences in solvent molecular size, where larger DMF molecules are able to separate cation and anion molecules over greater distances, yielding more efficient ion dissociation and greater solubility. The development of solvent orthogonality with each of the salts is an enticing prospect that could allow consecutive solution depositions of salts to fabricate potential cascade (three or more active layers with HOMO-LUMO offsets favoring exciton dissociation and charge transfer at each interface)^19^ or multi-junction^20^ architectures with different cations for complementary NIR optical absorption.

In addition to the altered electrochemical properties of the organic molecular salts, the anion exchange pathway provides an opportunity to compose safer and more stable chemicals by design. Approaches to reduce toxicity include avoiding toxicophores and substitutions using isosteres which have similar electronic characteristics but lower toxicity^21^. Design rules to hinder degradation should favor halogens, mostly chlorine and fluorine, chain branching, and nitrogen that are utilized here^22^.

## Conclusion

In summary, we have demonstrated organic molecular salts utilizing a range of phenyl borate and carborane anions for wavelength-selective photovoltaic applications. The phenyl borates demonstrate the full breadth of the effects of electron withdrawing functional groups on the density of states in a NIR-selective heptamethine salt, while the carboranes introduce a potential pathway toward highly stable organic wavelength-selective photovoltaics. This study explores the performance and physical effects of the new anions while relating the latter to previously demonstrated anions with different molecular structures. The ability to tune the interface gap, surface energy, and solubility through anion exchange offers a promising pathway toward tunable, efficient, safe, and stable NIR-selective transparent photovoltaics.

## Methods

### Synthesis of Cy-PhB, Cy-ClPhB, Cy-FPhB, and Cy-TFM

2-[2-[2-chloro-3-[2-(1,3-dihydro-3,3-dimethyl-1ethyl-2H-benz[e]indol-2-ylidene)ethylidene]−1-cylohexen-1-yl]-ethenyl]−3,3dimethyl-1-ethyl-1H-benz[e]indolium iodide (CyI, American Dye Source), sodium tetraphenylborate (NaPhB, Sigma Aldrich), potassium tetrakis(4-chlorophenyl)borate (KClPhB, TCI), sodium tetrakis(4-fluorophenyl)borate (NaFPhB, TCI), sodium tetrakis[3,5-bis(trifluoromethyl)borate (NaTFM, Sigma Aldrich) and potassium tetrakis(pentafluorophenyl)borate (KTPFB, Boulder Scientific Company) were used as received. The synthesis of CyTPFB has been reported previously^2^. Equimolar concentrations of Cy-I and the phenyl borate salt (Na-PhB, K-ClPhB, Na-FPhB, Na-TFM, or K-TPFB) were dissolved and stirred together at ~10 mg/ml in a 5:1 MeOH:dichloromethane (DCM) solution. The reaction proceeded in under five minutes, resulting in a solid precipitate that was then vacuum filtered and washed with MeOH. The one exception was NaTFM and CyI which did not precipitate, and the reaction solvent was rotoevaporated at 55 °C under vacuum from a diaphragm pump. The evaporating flask was washed with H_2_O to collect the crude product. All crude products were filtered through a plug of silica using DCM as the eluent. Verification of cations and anions and ion purity assessment were performed using a Waters Xevo G2-XS QToF mass spectrometer coupled to a Waters Acquity ultra-high performance liquid chromatography (UPLC) system. Cations were analyzed in positive ion mode electrospray ionization (ESI), and anions were analyzed in negative ion mode ESI. Solutions were prepared in acetonitrile and directly injected for 2 minutes using an eluent of 50:50 water:acetonitrile. Mass spectra were acquired using a dynamic range extension over m/z 50 to 1,500, with mass resolution (M/ΔM, full width-half maximum) of approximately 20,000. Other parameters include capillary voltage of 2 kV, desolvation temperature of 350 °C, source temperature of 100 °C, cone gas (N_2_) at 0 L/h, and desolvation gas (N_2_) at 400 L/h. Solutions with concentrations from 1–200 nM were prepared for CyI, NaPhB, KClPhB, NaFPhB, NaTFM, and KTPFB in acetonitrile to generate standard reference curves. The ion purity was found to be: > 99% (CyPhB), > 98% (CyClPhB), > 99% (CyFPhB), > 99% (CyTPFB), and > 98% (CyTFM).

### Synthesis of Cy-CBH and Cy-CoCB

CyI, cesium carborane (CsCBH, Strem), and sodium cobalticarborane (NaCoCB, Sigma Aldrich) were used as received. CyI and either CsCBH or NaCoCB were dissolved in equimolar concentrations at ~10 mg/ml in methanol (MeOH) and stirred for 5 minutes. The precipitate was collected using vacuum filtration and washed with MeOH. The crude product was re-dissolved in DCM at ~10 mg/ml and filtered through a plug of silica with DCM eluent. Reactants dissolved and stirred together in MeOH with immediate precipitation (<5 min.). Purity was verified using mass spectrometry as described above. Solutions with concentrations from 1–200 nM were prepared for CsCBH, CyCBH, NaCoCB and CyCoCB in acetonitrile to generate standard reference curves. The ion purity was measured to be > 99% for both CyCBH and CyCoCB, respectively.

### Synthesis of Cy_2_FCB

CyI and cesium dodecafluoro-closo-dodecaborate (CsFCB, Sigma Aldrich, used as received) were dissolved in equimolar concentrations in acetonitrile (~10 mg/ml) and stirred together for ten minutes. The precipitate was collected using vacuum filtration and washed with MeOH until the filtrate ran clear. The product was then Soxhlet filtered for five hours using MeOH. Purity was verified using Mass Spectrometry (Waters Xevo G2-XS quadrupole time-of-flight) with direct injection with 1–200 nM solutions of the reactants and products prepared in acetonitrile. The ion purity was found to be 92% for Cy-FCB.

### Solar Cell Device Fabrication and Testing

Pre-patterned ITO substrates (Xin Yan) were sequentially sonicated in soap, deionized water, and acetone before being rinsed in boiling isopropanol and treated in oxygen plasma for 3 minutes per step. MoO_3_ (Alfa Aesar) was thermally deposited under vacuum (base pressure of 3 × 10^−6^ Torr) at 0.1 nm/s. Cy powders were massed in air and dissolved in DMF or CB in nitrogen. Cy films were spincoated in nitrogen over MoO_3_ at 4000 rpm for 20 seconds. C_60_ (MER Corp.), BCP (Luminescence Technology, Inc.), and Ag (Kurt J Lesker Co.) were sequentially thermally deposited at 0.1 nm/s. *J-V* curves on 5.2mm^2^ devices were acquired under illumination from a Xe arc lamp with intensity calibrated to approximately 1-sun with a NREL-calibrated Si reference cell with KG5 filter. *EQE* measurements were performed under monochromated light from a tungsten halogen lamp chopped at 200 Hz and calibrated with a Newport-calibrated Si diode. Individual layer thicknesses were measured with variable angle spectroscopic ellipsometry (J. A. Wollam) on Si substrates.

### Contact Angle and Solubility Measurement

Contact angles on salt films were measured with a KRÜSS DSA-100 drop shape analyzer and surface energies were calculated using the Fowkes method^23^. Solubilities in CB and DMF were measured by adding solvents to vials with 2 mg of solute until particles were visibly dissolved, while those in water were calculated utilizing the Beer-Lambert law from UV/VIS transmission scans of solutions with known attenuation coefficients.

### Surface roughness and energetic characterization

AFM data was measured in contact mode for *ex situ* roughness characterization for salt films deposited on Si substrates from 2 mg/ml and 12 mg/ml solutions. Cross-sectional SEM data was measured on salt/C_60_ bilayers deposited on Si that was subsequently cleaved. UPS data was measured with He I radiation (21.2 eV) on salt films deposited on unpatterned ITO/MoO_3_ from 12 mg/ml solutions.

## Electronic supplementary material

Supplemental Information
