# Supplementary material for: Anions for Near-Infrared Selective Organic Salt Photovoltaics
Source: Sci Rep. 2017 Nov 27;7:16399. doi: 10.1038/s41598-017-16539-3 (PMC5703893; doi:10.1038/s41598-017-16539-3)
Supplement: Supplementary file 1 — Supplemental Information [file 41598_2017_16539_MOESM1_ESM.pdf]

Anions for Near-Infrared Selective Organic Salt Photovoltaics

Christopher J. Traverse,[1] Margaret Young,[1] John Suddard-Bangsund,[1] Tyler Patrick,[1] Matthew Bates,[1] Pei Chen,[1] Brian Wingate,[2] Sophia Y. Lunt,[3,1] Annick Anctil,[4] Richard R. Lunt[1, 5, *]

[1] *Department of Chemical Engineering and Materials Science, Michigan State University, East Lansing, MI 48824 USA*[2] *Department of Mechanical Engineering, Michigan State University, East Lansing, MI 48824 USA*

[3] *Department of Biochemistry, Michigan State University, East Lansing, MI 48824 USA*

[4] *Department of Civil and Environmental Engineering, Michigan State University, East Lansing, MI 48824 USA*

[5] *Department of Physics and Astronomy, Michigan State University, East Lansing, MI 48824 USA*

[*] rlunt@msu.edu

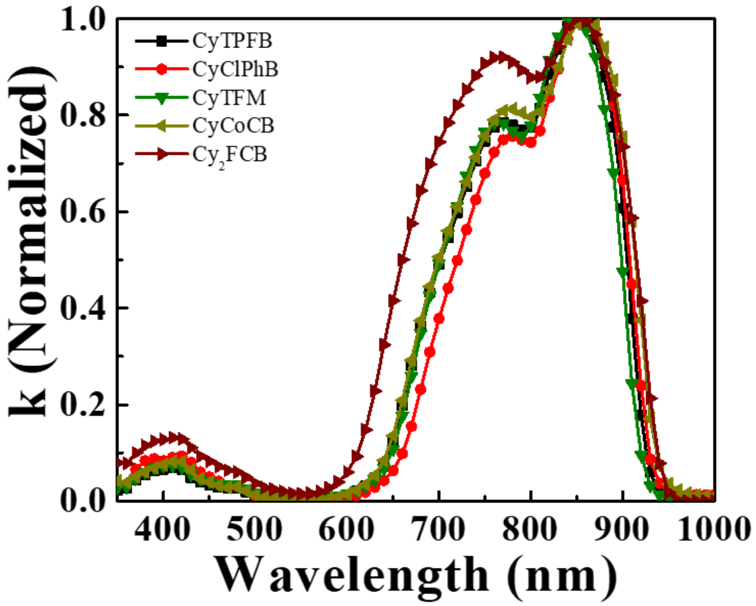

Figure S1. Extinction coefficients for Cy^+ cation with selected anions. Normalized extinction coefficients representing optical absorption for CyTPFB, CyClPhB, CyTFM, CyCoCB, and Cy_2FCB salt films measured via spectroscopic ellipsometry highlighting little change in the bandgap or absorption spectra with changes in the anion.

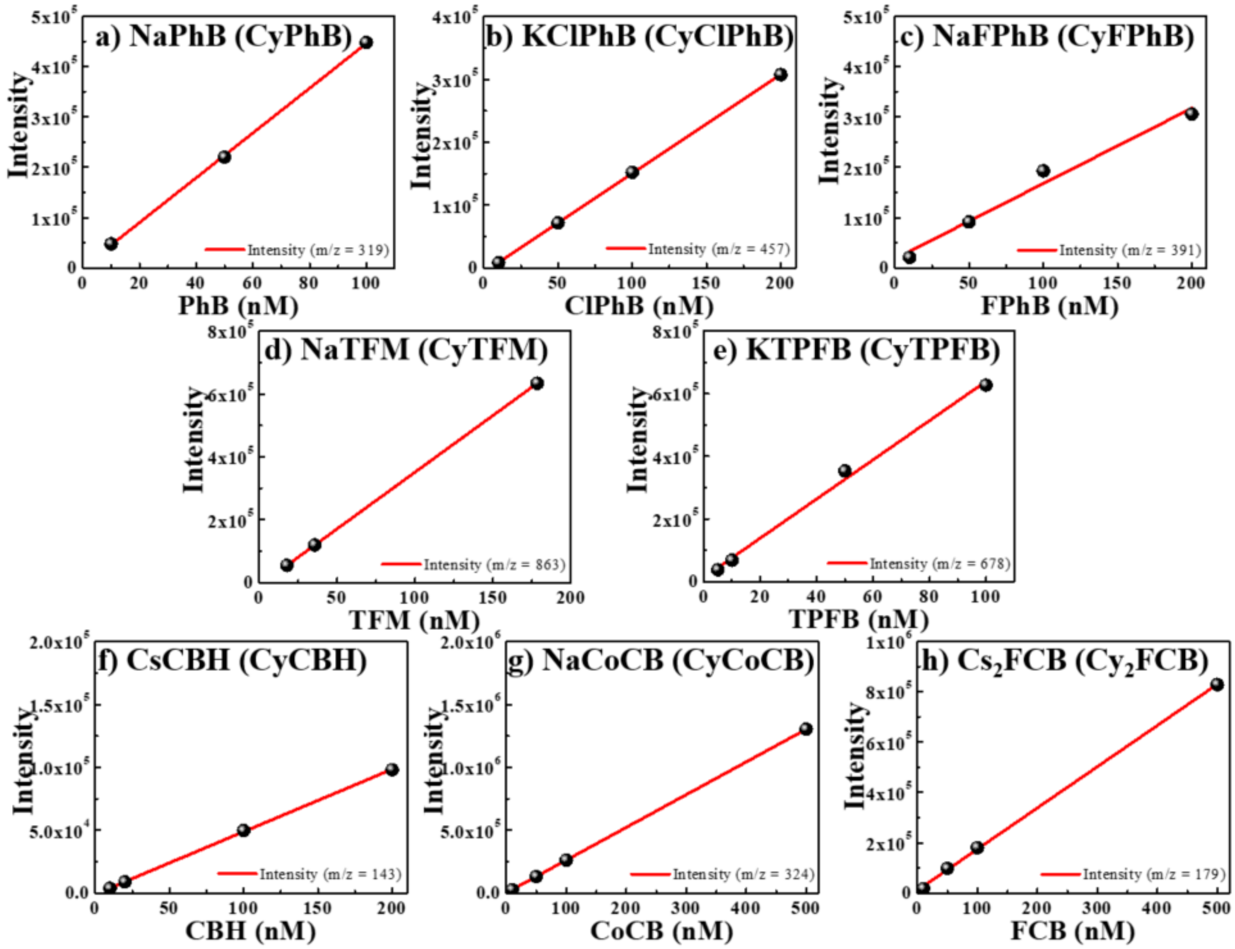

Figure S2. Mass spectrometry calibration. Calibration curves of intensity for *m/z* = 319 (PhB) signal for NaPhB standard (a), *m/z* = 457 (ClPhB) signal for KClPhB standard (b), *m/z* = 391 (FPhB) signal for NaFPhB (c), *m/z* = 863 (TFM) signal for NaTFM standard (d), *m/z* = 678 (TPFB) signal for KTPFB standard (e), *m/z* = 143 (CBH) signal for CsCBH standard (f), *m/z* = 324 (CoCB) signal for NaCoCB standard (g), and *m/z* = 179 (FCB) signal for Cs_2FCB standard (f).

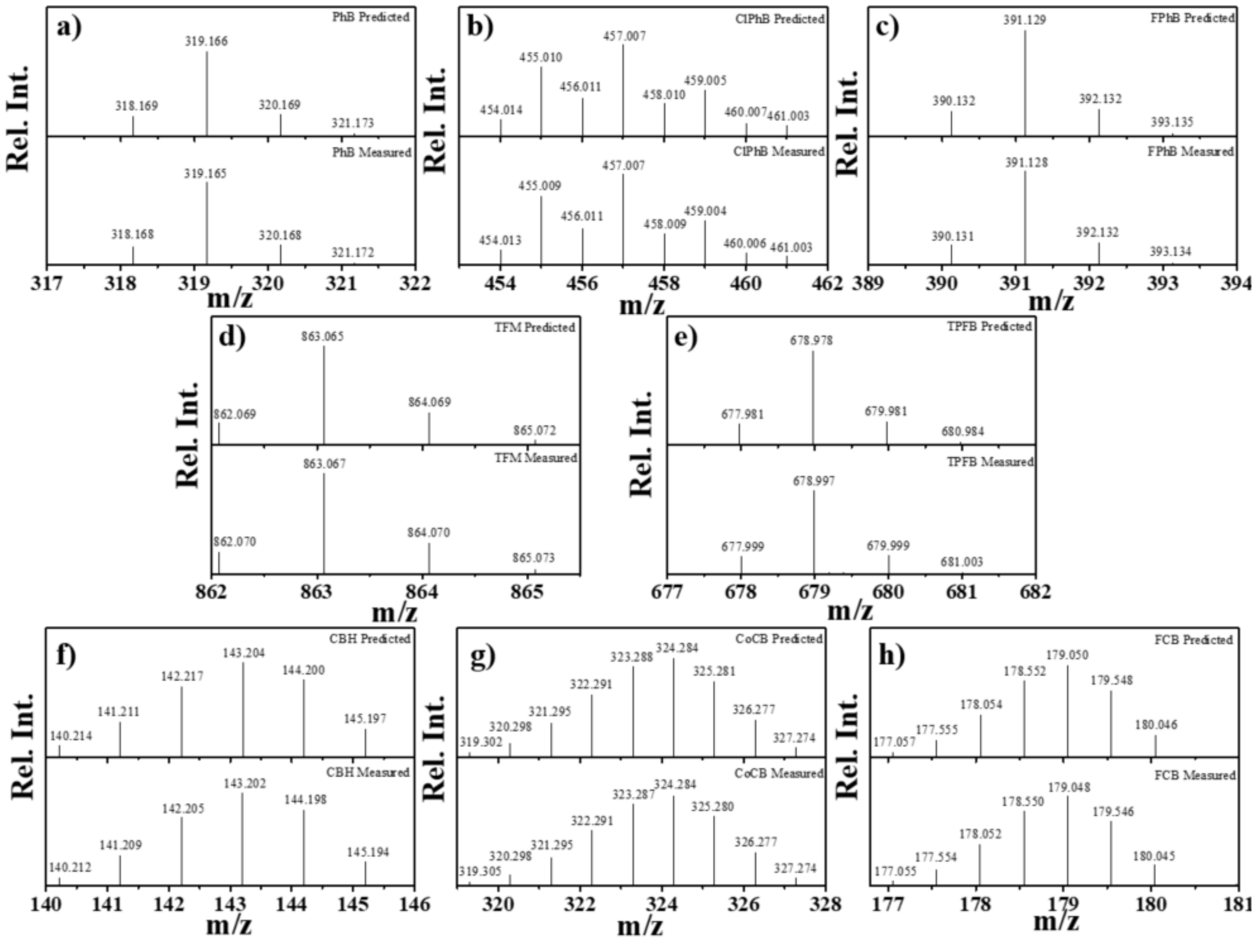

Figure S3. Mass spectra. High resolution mass spectrometry verification in negative mode electrospray ionization for CyPhB (a), CyClPhB (b), CyFPhB (c), CyTFM (d), CyTPFB (e), CyCBH (f), CyCoCB (g), and Cy_2FCB (h). Predicted isotopic abundance peaks for each compound were generated using the Isotope Model tool in MassLynx software.

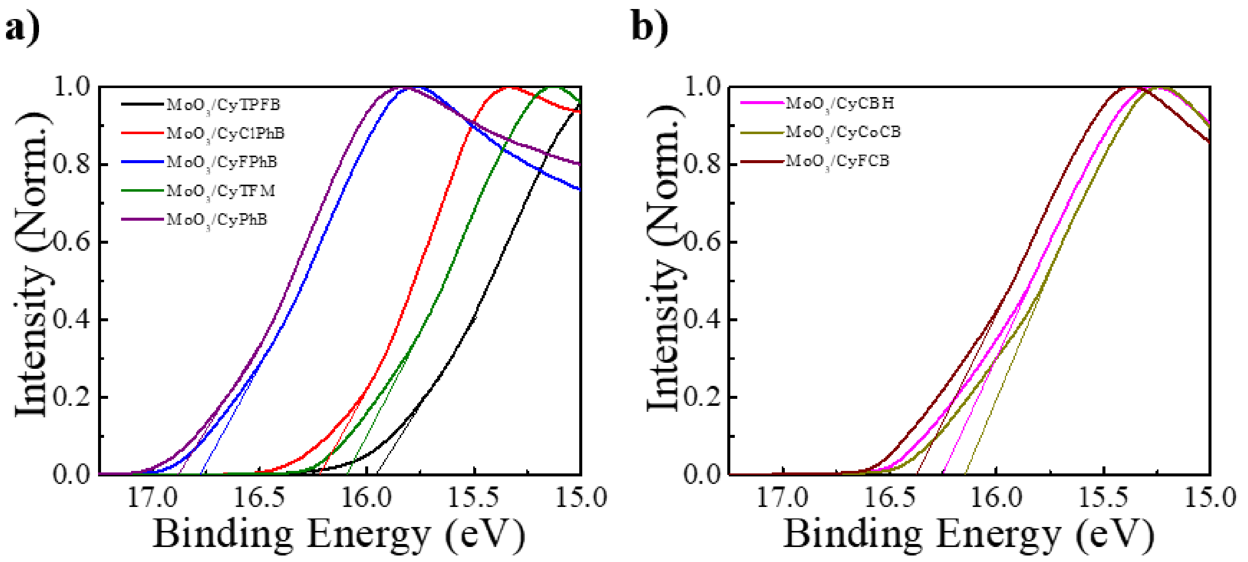

Figure S4. Measured work functions. Ultraviolet photoelectron spectroscopy (UPS) data for salts with (a) phenyl borate and (b) carborane anions.

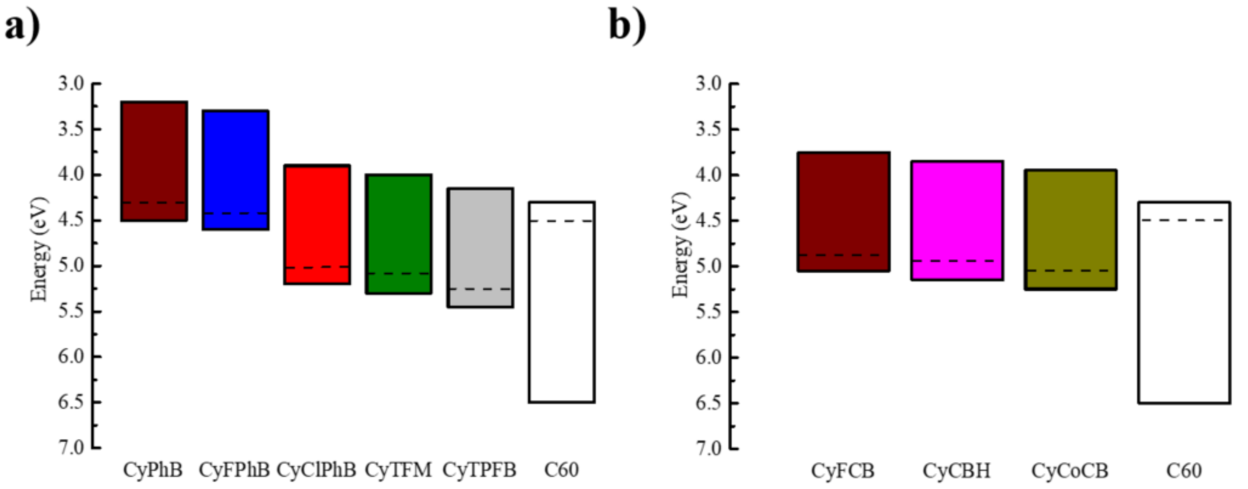

Figure S5. Salt energy levels. Energy diagrams measured for salts with phenyl borate (a) and carborane (b) anions. HOMOs are estimated as a 0.2 eV offset below the measured work functions while LUMOs are approximated as the HOMO level plus the optical excitonic gap (1.3 eV). The HOMO trends follow the observed V_{oc} trends with respect to anion.

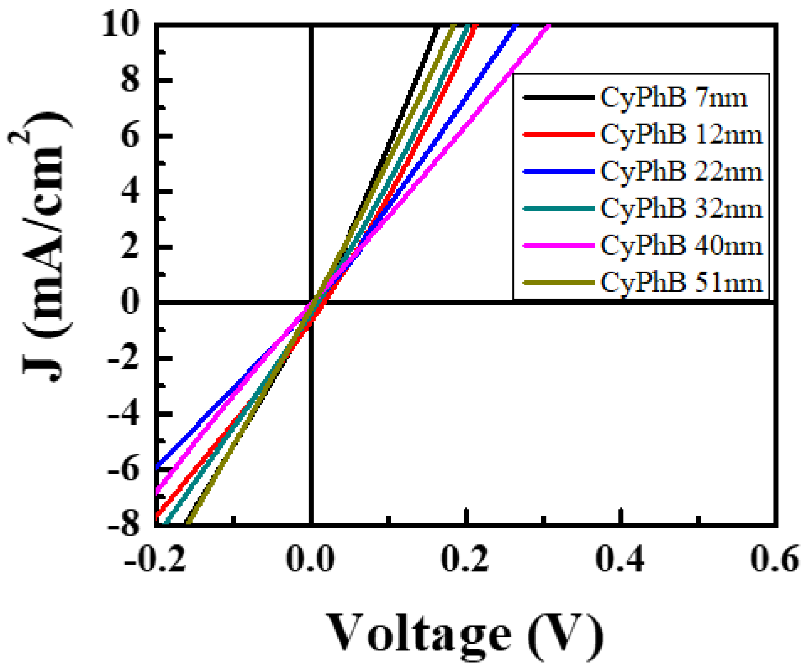

Figure S6. CyPhB Thickness Dependence. Current (*J*)-voltage (*V*) characteristics for devices with several CyPhB donor layer thicknesses. Devices exhibit small photocurrent and photovoltage and high leakage current due to a nearly negligible interface gap between CyPhB and C_{60}.

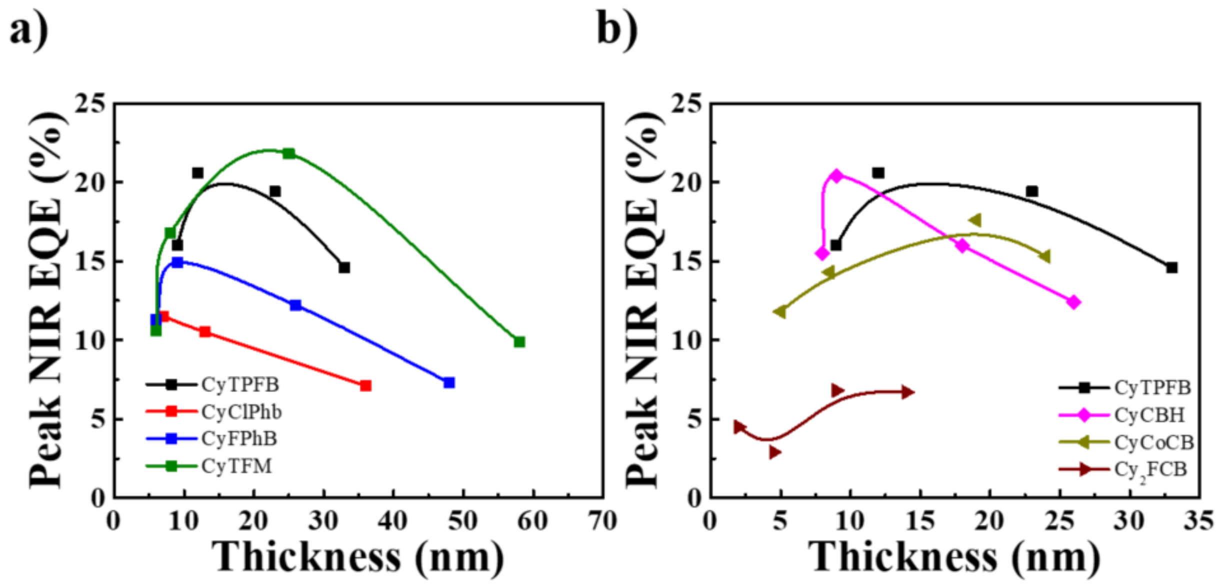

Figure S7. Near-infrared EQE thickness dependence. NIR *EQE* peaks for phenyl borate (a) and carborane (b) anion salt devices as a function of salt layer thickness. The solid lines are guides to the eye.

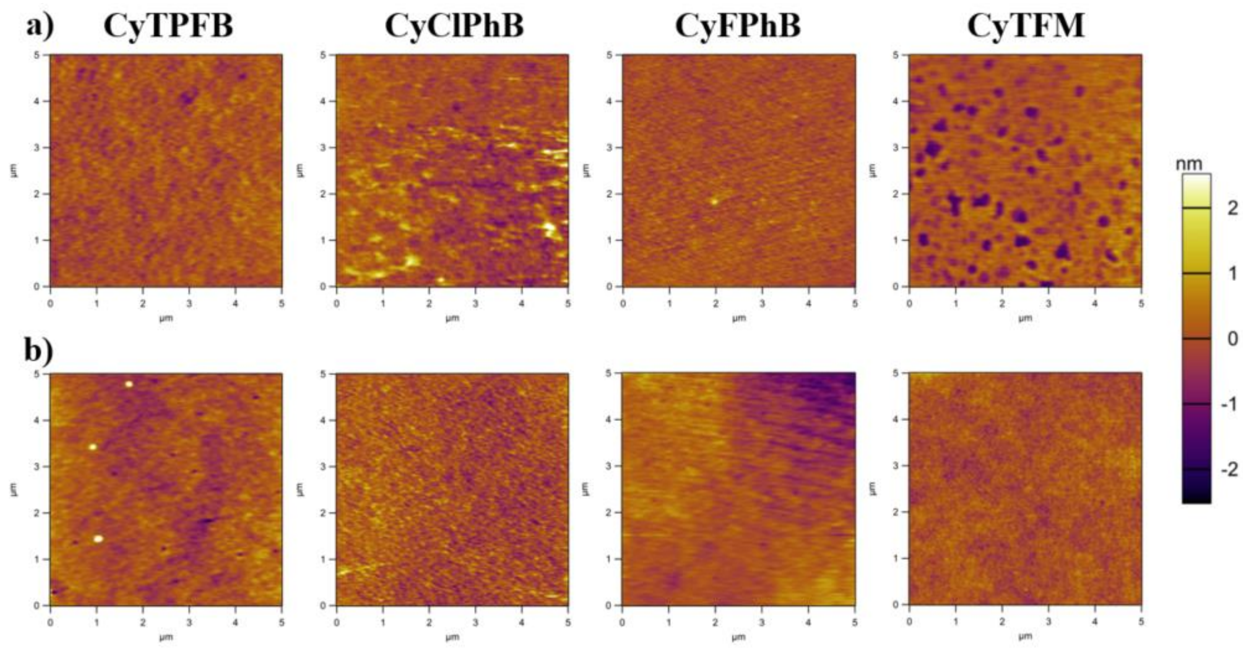

Figure S8. Salt film morphologies. Atomic force microscopy (AFM) data taken from isolated films of CyTPFB, CyClPhB, CyFPhB, and CyTFM deposited over Si from 2 mg/ml (a) and 12 mg/ml (b) solutions. The roughness scale used for all images is shown at the right. Salt films are smooth across the two thickness extremes, which suggests that roughness does not play a large role in the voltage changes observed with increasing thicknesses of CyClPhB, CyFPhB, and CyTFM.

Salt	2mg/ml Roughness (nm)	12mg/ml Roughness (nm)
CyTPFB	0.26 ± 0.01	0.36 ± 0.04
CyClPhB	0.44 ± 0.03	0.40 ± 0.03
CyFPhB	0.25 ± 0.01	0.4 ± 0.1
CyTFM	0.41 ± 0.03	0.27 ± 0.01

Table S1. Measured roughness. Calculated RMS roughness values from the AFM data in Figure S8.

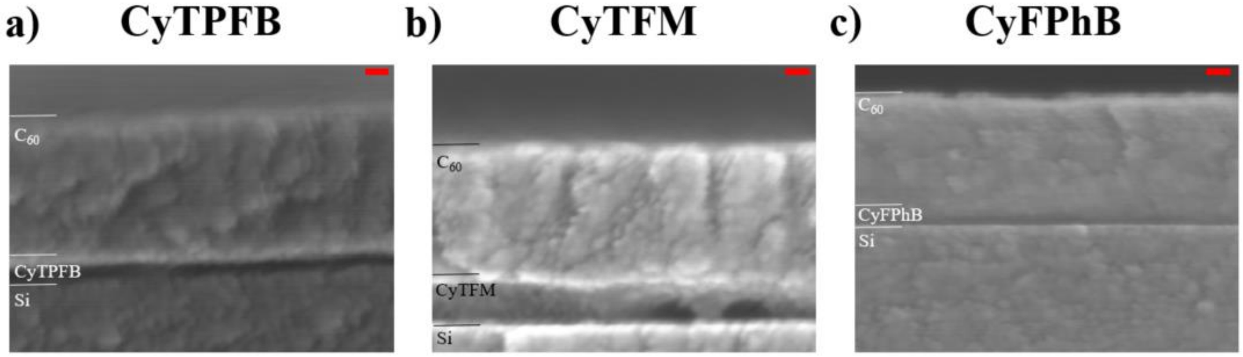

Figure S9. Interfacial morphologies. Cross-sectional scanning electron microscopy (SEM) images taken from (a) CyTPFB, (b) CyTFM, and (c) CyFPhB / C_{60} bilayers deposited over Si (from bottom to top: Si / Salt / C_{60}). The bright bands (~2 nm) between the salt and C_{60} layers suggests possible molecular intermixing resulting in higher resistivity and slight charging. The red scale bars at the top right corner of each image indicate a distance of 10 nm.

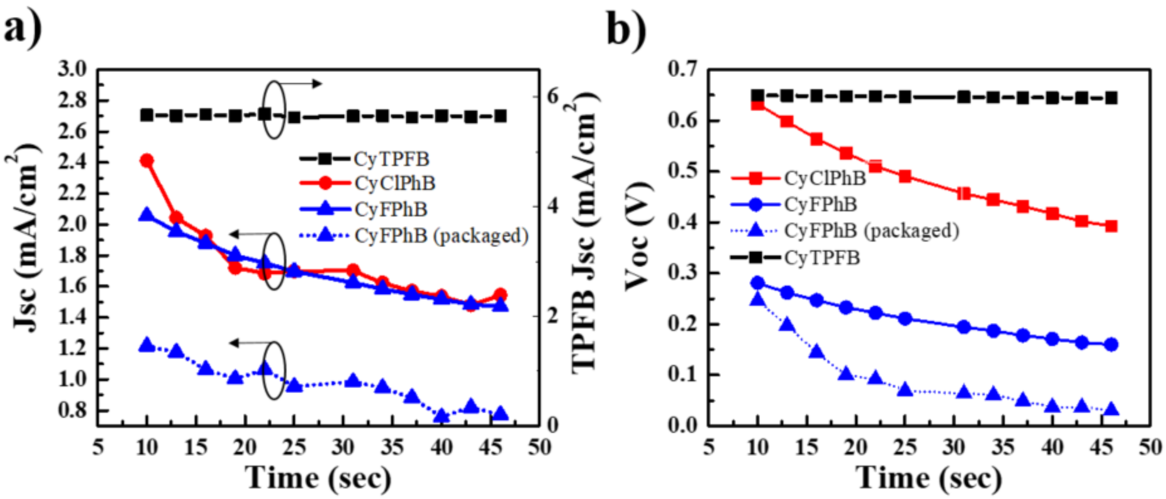

Figure S10. Rapid photodegradation of CyFPhB and CyClPhB. J_{sc} (a) and V_{oc} (b) parameters for CyClPhB, CyFPhB, and CyTPFB devices extracted from *J*-*V* curves taken over 50 seconds under illumination from a Xe arc lamp. "Packaged" CyFPhB devices were sealed under UV-cured epoxy in a nitrogen environment prior to illumination and testing. While many of the salts are highly stable, the CyFPhB and CyClPhB stood out as being particularly unstable.
